# Impact of Hyperbaric Oxygen Therapy on Subsequent Neurological Sequelae Following Carbon Monoxide Poisoning

**DOI:** 10.3390/jcm7100349

**Published:** 2018-10-13

**Authors:** Chien-Cheng Huang, Chung-Han Ho, Yi-Chen Chen, Chien-Chin Hsu, Yi-Fong Wang, Hung-Jung Lin, Jhi-Joung Wang, How-Ran Guo

**Affiliations:** 1 Department of Emergency Medicine, Chi-Mei Medical Center, Tainan 710, Taiwan; chienchenghuang@yahoo.com.tw (C.-C.H.); nych2525@gmail.com (C.-C.H.); hjlin52@gmail.com (H.-J.L.); 2 Department of Environmental and Occupational Health, College of Medicine, National Cheng Kung University, Tainan 701, Taiwan; 3 Department of Senior Services, Southern Taiwan University of Science and Technology, Tainan 71005, Taiwan; 4 Department of Medical Research, Chi Mei Medical Center, Tainan 710, Taiwan; ho.c.hank@gmail.com (C.-H.H.); laura751111986@hotmail.com (Y.-C.C.); 400002@mail.chimei.org.tw (J.-J.W.); 5 Department of Hospital and Health Care Administration, Chia Nan University of Pharmacy and Science, Tainan 71710, Taiwan; 6 Department of Biotechnology, Southern Taiwan University of Science and Technology, Tainan 71005, Taiwan; 7 Department of Leisure, Recreation and Tourism Management, Southern Taiwan University of Science and Technology, Tainan 71005, Taiwan; yfwang@stust.edu.tw; 8 Department of Emergency Medicine, Taipei Medical University, Taipei 110, Taiwan; 9 Department of Occupational and Environmental Medicine, National Cheng Kung University Hospital, Tainan 704, Taiwan; 10 Occupational Safety, Health, and Health Research Center, National Cheng Kung University Hospital, Tainan 704, Taiwan

**Keywords:** carbon monoxide poisoning, hyperbaric oxygen therapy, neurological sequelae

## Abstract

The purpose of this study was to evaluate the effects of hyperbaric oxygen therapy (HBOT) on reducing neurological sequelae (NS) in patients with carbon monoxide poisoning (COP). Using a nationwide database of insurance claims in Taiwan, we conducted a population-based cohort study to identify 24,046 patients with COP diagnosed between 1999 and 2012, including 6793 (28.2%) patients who received HBOT and 17,253 (71.8%) patients who did not. We followed the two cohorts of patients and compared the occurrence of NS. The two cohorts had similar sex ratios, but patients who received HBOT were younger (34.8 ± 14.8 vs. 36.1 ± 17.2 years, *p* < 0.001). Patients who received HBOT had a higher risk for NS (adjusted hazard ratio [AHR]: 1.4; 95% confidence interval [CI]: 1.4–1.5), after adjusting for age, sex, underlying comorbidities (hypertension, diabetes, chronic obstructive pulmonary disease, hyperlipidemia, malignancy, coronary artery disease, congestive heart failure, liver disease, renal disease, connective tissue disease, human immunodeficiency virus [HIV] infection, and alcoholism), monthly income, suicide, drug poisoning, and acute respiratory failure. We observed similar findings when we stratified the patients by age, sex, underlying comorbidities, and monthly income. The increased risk was most prominent in the first 2 weeks (AHR: 2.4; 95% CI: 2.1–2.7) and remained significant up to 6 months later (AHR: 1.6; 95% CI: 1.4–1.7). The risk for NS was higher in patients with COP who received HBOT than in those who did not, even after considering the possible impact of longer observation periods on survivors. Further studies that included the potential confounding factors we did not measure are needed to confirm findings in this study.

## 1. Introduction

Carbon monoxide (CO) is a toxic product from the incomplete combustion of carbon-based compounds by water heaters, charcoal burning, motor vehicles, gas-powered furnaces, and other domestic or industrial fuel-burning processes [1,2,3]. Even a small amount of CO can cause severe tissue hypoxia because the affinity of CO for hemoglobin is about 250 times greater than that for oxygen [4]. In the United States, carbon monoxide poisoning (COP) is responsible for an estimated 50,000 emergency department visits annually and is one of the leading causes of death due to poisoning [5,6]. In addition to accidental poisoning, COP by burning charcoal has become one of the most popular methods for suicide in recent years [7]. In Taiwan, the incidence of suicidal COP by burning charcoal increased by 25-fold between 1999 and 2009 [7]. 

The suggested treatment for COP is administration of 100% normobaricoxygen (NBO) by non-rebreathing mask or endotracheal intubation [6,8]. The additional benefit of hyperbaric oxygen therapy (HBOT) for reducing subsequent neurological sequelae (NS) has been a subject of debates [6,9,10,11]. A double-blind randomized trial in 2002 reported that HBOT can reduce the risk for NS at 6 weeks and 12 months, which is the most important evidence supporting HBOT [12]. However, a meta-analysis in 2011 showed that existing randomized trials failed to prove HBOT’s effect of reducing NS [9]. On the other hand, our previous study found that HBOT was associated with a substantial reduction in the mortality after COP [13]. Therefore, we conducted a nationwide population-based cohort study in Taiwan to clarify this issue.

## 2. Experimental Section

### 2.1. Data Source

We used the Nationwide Poisoning Database (NPD), which is a sub-database from the Taiwan National Health Insurance Research Database (NHIRD), for this study. The NPD contains all the poisoning cases, including COP, between 1999 and 2013 in Taiwan. The NHIRD encompasses data from the Taiwan National Health Insurance program, which comprises nearly 100% of the population including foreigners in this country [14]. The database of this program contains registration files and original claim data for reimbursement [14]. Large computerized databases derived from this system by the National Health Insurance Administration, Ministry of Health and Welfare, Taiwan, and maintained by the National Health Research Institutes, Taiwan, are provided to scientists in Taiwan for research purposes [14].

### 2.2. Identification of Patients with COP

**Figure 1:**
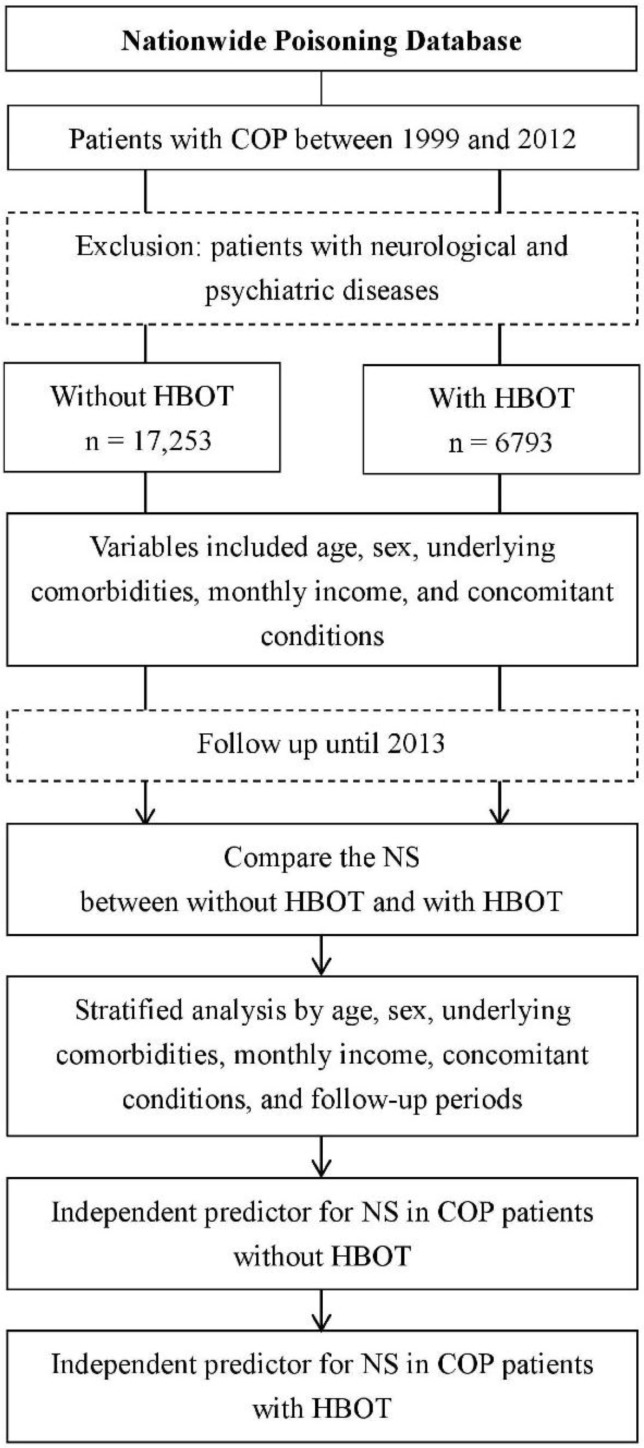
Flowchart of this study. COP, carbon monoxide poisoning; HBOT, hyperbaric oxygen therapy; NS, neurological sequelae.

We identified all the patients with COP by the International Classification of Diseases, Ninth Revision, Clinical Modification (ICD-9-CM): 986, E868, E952, or E982 in either hospitalization or emergency department care as one of the main diagnoses from the NPD between 1999 and 2012 (Figure 1). The diagnosis of COP in Taiwan is generally based on documented CO exposure (elevated carboxyhemoglobin [COHb] levels or ambient CO concentrations) and any of the following symptoms: headache, malaise, fatigue, forgetfulness, dizziness, loss of consciousness, confusion, visual disturbances, nausea, vomiting, cardiac ischemia, or metabolic acidosis (a calculated base excess level < −2.0 mmol/L or a blood lactate level >2.5 mmol/L). If the COHb level was <10%, the patient was eligible only if COP was the only plausible diagnosis [3,4,15,16]. Regarding NS, we focused on any manifestations of impairments in concentration, language, or calculation, cognitive dysfunction, delirium, amnesia, Parkinson-like syndromes, gait disturbance, corticospinal tract signs of damage, urine and fecal incontinence, vestibular problems, depression, anxiety, or affective changes [15]. Therefore, we defined the criteria of NS as including the diagnosis of degenerative diseases of the central nervous system (ICD-9-CM: 290, 331–337), psychiatric diseases (ICD-9-CM: 293–298, 300), or other diseases of the nervous system (ICD-9-CM: 338–352) in at least one hospitalization or three occurrences of ambulatory care after COP. Because the claim diagnosis may be incorrect in the ambulatory care, we used at least three occurrences of ambulatory care to confirm the diagnosis, which has been widely used and confirmed in studies based on the NHIRD [17]. Patients who had the same diagnosis of neurological or psychiatric diseases before and after COP were excluded to ensure that the onset of NS was after the poisoning.

### 2.3. Definitions of Variables

In addition to COP, we included the following variables in the analysis: age, sex, underlying comorbidities, monthly income, and concomitant conditions. Age was classified into the following subgroups: <20, 20–34, 35–49, 50–64, and ≥65 years. Underlying comorbidities were defined as at least one hospitalization or three occurrences of ambulatory care of the diagnoses before COP: hypertension (ICD-9-CM 401–405), diabetes (ICD-9-CM 250), chronic obstructive pulmonary disease (COPD; ICD-9-CM 496), hyperlipidemia (ICD-9-CM 272), malignancy (ICD-9-CM 140–208), coronary artery disease (ICD-9-CM 410–414), congestive heart failure (ICD-9-CM 428), liver disease (ICD-9-CM 570–576), renal disease (ICD-9-CM 580–593), connective tissue disease (ICD-9-CM 710), HIV infection (ICD-9-CM 042, 079.53, V08), and alcoholism (ICD-9-CM 291, 303, 3050, 3575, 4255, 5353, 5710–5713, or V113). Monthly income was classified as <19,999, 20,000–39,999, and ≥40,000 New Taiwan Dollars (NTD) [18]. Concomitant conditions were defined as suicide (management codes 94.0 or 94.1, or ICD-9-CM E950–E959), drug poisoning (ICD-9-CM 960–989, exclusion of 986), acute respiratory failure (ICD-9-CM 518.81 or 518.84, or management codes 960, 9601, 9602, 9603, 9604, 9605, 9390, 9391, or 311), acute myocardial injury (ICD-9-CM 410), acute hepatitis (ICD-9-CM 573.3), and acute renal failure (ICD-9-CM 584 or management code 339.5) during the same episode of COP. The references for ICD-9-CM and management code can be found in the websites of Taiwan National Health Insurance Administration [19,20].

### 2.4. Comparison of the Risk for NS Between the Two Cohorts and Independent Predictors for NS

We compared the risk for NS between the two cohorts until 2013. Stratified analyses by age, sex, underlying comorbidities, monthly income, suicide, drug poisoning, acute respiratory failure, and follow-up periods were also performed to evaluate the presence of effect modification. The independent predictors for NS in the two cohorts were also investigated individually.

### 2.5. Ethics Statement

The Institutional Review Board at Chi-Mei Medical Center approved this study, which was conducted in strict accordance with the Declaration of Helsinki. Given that the NPD contains de-identified information, the need for informed consent from the patients was waived as it did not affect the rights or welfare of the patients.

### 2.6. Data Analysis

Independent t-tests for continuous variables and chi-square tests for categorical variables were used to evaluate the differences in demographic characteristics, underlying comorbidities, monthly income, and concomitant conditions between the two cohorts. Cox proportional hazard regression and Kaplan–Meier method with log-rank test were performed to compare the incidence of NS between the two cohorts. We performed additional Poisson regression analysis to evaluate the risk for NS stratified by follow-up periods between the two cohorts. Cox proportional hazard regression analysis was conducted to investigate the independent predictors for NS in the two cohorts individually. We also used propensity score matching and Cox proportional hazard regression analysis to validate the result. Because a longer survival period leads to a higher probability of developing NS, we conducted an additional analysis combining NS and death as the outcome to evaluate the possible effect. All the analyses were performed using SAS 9.4 for Windows (SAS Institute, Cary, NC, USA) at a two-tailed significance level of 0.05.

## 3. Results

A total of 24,046 COP patients, including 6793 (28.2%) patients who received HBOT and 17,253 (71.8%) patients who did not, were identified in this study (Table 1). Patients who received HBOT were younger than those who did not (34.8 ± 14.8 years vs. 36.1 ± 17.2 years, *p* < 0.001). The sex ratio and monthly income did not differ between the two cohorts. Patients who received HBOT had lower prevalence of underlying comorbidities of hypertension, diabetes, hyperlipidemia, malignancy, coronary artery disease, congestive heart failure, COPD, liver disease, and alcoholism, but not other comorbidities. During the COP episode, patients who received HBOT presented higher percentages of suicide (31.2% vs. 15.3%), acute respiratory failure (9.7% vs. 6.2%), and acute renal failure (1.9% vs. 1.0%) than the patients who did not. 

**Figure 2:**
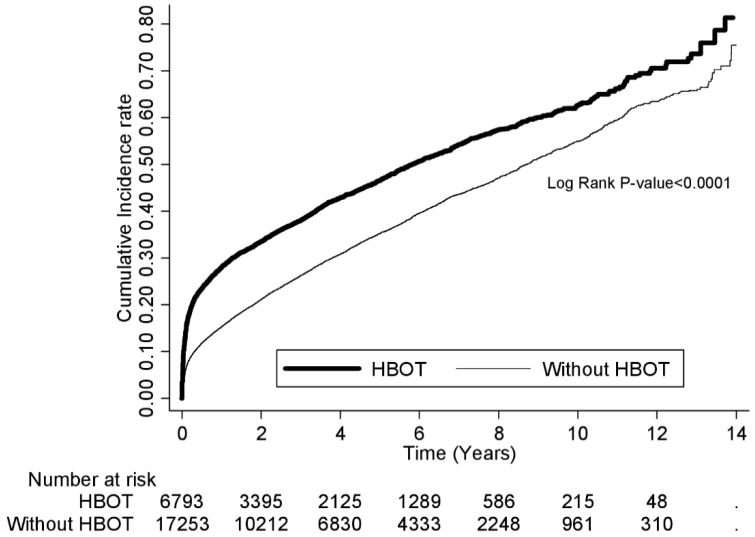
Follow-up risk for NS between COP patients with and without HBOT by Kaplan–Meier method with log-rank test.

Cox proportional hazard regression analysis showed a higher risk for NS among the patients who received HBOT than the patients who did not (adjusted hazard ratio [AHR]: 1.4; 95% confidence interval [CI]: 1.4–1.5) after adjusting for age, sex, monthly income, suicide, drug poisoning, acute respiratory failure and underlying comorbidity of hypertension, diabetes, COPD, hyperlipidemia, malignancy, coronary artery disease, congestive heart failure, liver disease, renal disease, connective tissue disease, HIV infection, and alcoholism (Table 2). Stratified analyses showed that the increased risk for NS in the patients who received HBOT was also significant in the five age subgroups, two sex subgroups, all subgroups defined by the underlying comorbidities except for congestive heart failure, three subgroups of monthly income, and subgroups defined by concomitant conditions of suicide, drug poisoning, and acute respiratory failure. During the follow-up period, patients who received HBOT presented the highest risk for NS in the first 2 weeks after COP (AHR: 2.4; 95% CI: 2.1–2.7), followed by 2–4 weeks (AHR: 1.6; 95% CI: 1.4–1.9) and 1–6 months (AHR: 1.6; 95% CI: 1.4–1.7). Poisson regression analysis showed similar findings: the incidence rate ratio (IRR) was the highest in 2 weeks (2.6; 95% CI: 2.3–2.9), followed by 2–4 weeks (IRR: 2.3; 95% CI: 2.0–2.7) and 1–6 months (IRR: 1.8; 95% CI: 1.7–2.0). Kaplan–Meier method with log-rank test also demonstrated a higher risk for NS in the patients who received HBOT than in the patients who did not (*p* < 0.0001; Figure 2). Propensity score matching and Cox proportional hazard regression analysis also showed patients who received HBOT had a higher risk for NS than those without (AHR: 1.4; 95% CI: 1.3–1.5) (Supplement Table 1 and Table 2). In the additional analysis combining NS and death as the outcome, patients who received HBOT still had a higher risk than those who did not, but the difference became smaller (AHR: 1.2; 95% CI: 1.1–1.2).

The incidence rates of degenerative diseases of the central nervous system, psychiatric diseases, and other diseases of the nervous system were 23.2, 87.8, and 57.6 per 1000 person-years in patients who received HBOT and 14.9, 59.3, and 34.9 per 1000 person-years in patients who did not receive HBOT, respectively (Table 2). The AHR observed in the analysis of each subgroup of NS was similar to that observed in the overall analysis. 

The independent predictors for NS in the patients who received HBOT were old age; male sex; low monthly income; underlying comorbidity of hypertension, malignancy, liver disease, and alcoholism; and concomitant conditions of suicide and acute respiratory failure (Table 3). Except for comorbidity of hypertension, malignancy, and liver disease, they are also independent predictors for NS in the patients who did not receive HBOT (Table 3). However, underlying comorbidity of COPD and hyperlipidemia also appeared to be independent predictors for NS in patients who did not receive HBOT.

## 4. Discussion

This study showed that the risk for NS in the patients who received HBOT was higher than that in the patients who did not, especially in the first 6 months after poisoning. The increased risk was also found in the subgroup analyses, including those by age, sex, underlying comorbidities except for congestive heart failure, monthly income, and concomitant conditions of suicide, drug poisoning, and acute respiratory failure. The common independent predictors for NS in the two cohorts were old age, male sex, low monthly income, underlying comorbidity of alcoholism, and concomitant conditions of suicide, and acute respiratory failure.

A meta-analysis conducted in 2011 showed that existing randomized controlled trials reported conflicting results regarding HBOT vs. NBO [9]. A prospective randomized controlled trial recruiting 385 patients with acute domestic COP showed that HBOT had no benefit in patients with transient loss of consciousness and resulted in poor outcome in comatose patients [21]. The results might have been affected by the lack of blinding, loss to follow-up (at least 14% at 1 month), and the difference in the proportion of coma cases before admission. Nevertheless, a double-blind randomized controlled trial reported that HBOT had no benefit and might even have worsened the outcome [22]. While one of our previous studies showed that COP was associated with increased long-term mortality [23], another showed that HBOT reduced long-term mortality [13]. Therefore, a possible explanation of the higher risk for NS in the patients who received HBOT was that the life-saving effect of HBOT is more prominent than its possible NS-reducing effect. In other words, HBOT might have saved the lives of some patients who were at higher risks for NS but would have passed away before developing NS if not for the HBOT. Our previous study found that HBOT was associated with an overall 36% reduction in the mortality (AHR: 0.74; 95% CI: 0.67–0.81) and that the effect was most prominent in the first 2 weeks (AHR: 0.51; 95% CI: 0.37–0.72) [13], which is also the period with the highest mortality in the current study. However, the additional analysis that combined NS and death as the outcome demonstrated that the AHR decreased to 1.2 but was still statistically significant (95% CI: 1.1–1.2), indicating the presence of other factors. Another possible factor is that patients with a higher risk for NS were prone to receive HBOT (Table 1) in this study. The general indications for HBOT are also risk factors for NS [24,25,26,27,28,29,30], and even though we have adjusted for age and comorbidities in the analyses, we were unable to adjust for all the other risk factors. As a results, patients who received HBOT had higher prevalence of having risk factors for NS in comparison with those who did not, and this would contribute to the higher incidence of NS associated with HBOT observed in this study.

The protocol for HBOT differs among studies, which might affect the outcome. The most well-known protocol for HBOT is three hyperbaric oxygen treatments including 3 atmosphere absolute (ATA) at first chamber session and 2 ATA at second and third chamber sessions within a 24 h period [12]. This protocol reduced the risk for NS at 6 weeks and 12 months after acute COP [12]. However, the double-blind randomized controlled trial might suffer from bias introduced during data analysis. The potential sources of the bias included the differences in the outcomes analyzed between the preliminary report and the final report, reassigning participants to different groups based on assumptions, and the premature termination of the trial [9]. In Taiwan, the following protocol is generally adopted, but variations exist due to the differences in facilities and staff: (1) first session within 6–24 h as soon as possible; (2) 2.5–3.0 ATA; (3) 60–120 min each session; (4) treatments of oxygen supply (non-rebreathing mask vs. endotracheal intubation) and chamber choice (monoplace vs. multiplace chamber) depending on the patient’s condition and medical resource; (5) one session each day; and (6) one to five sessions in total [13,30]. However, no studies have been conducted to compare the two protocols.

In our study, suicide was a predictor for NS in both patients who have received and not received HBOT, independent of other well-known risk factors. In the past 20 years, burning charcoal has become one of the most common methods of suicide in Taiwan and Hong Kong, contributing to a 20% increase in the overall suicide rate [31]. Patients attempting suicide might be more severely intoxicated, and thus might have worse prognosis. In the present study, low income predicted NS, which was similar to our previous finding that low income was an independent predictor of long-term mortality after COP [23]. Low income is associated with poor living environment and limited resources (such as CO detectors), which might contribute to the increased risk [32,33]. Furthermore, low income is associated with increased poor awareness of disease prevention, less and inconvenient access to medical care, and behavioral risk factors such as smoking [34,35], which might also contribute to this finding in the present study.

The independent predictors of NS differed between patients who have received and not received HBOT in our study. Specifically, while both COPD (AHR: 1.5; 95% CI: 1.1–2.1) and hyperlipidemia (AHR: 1.3; 95% CI: 1.1–1.5) were independent predictors of NS in patients who did not receive HBOT, they were associated with decreased AHRs (1.0(95% CI: 0.8–1.2) for COPD and 1.0(95% CI: 0.9–1.1) for hyperlipidemia) in patients who received HBOT.

This nationwide population-based study involved a large sample size to delineate a controversial issue in clinical practice. Nonetheless, our study had some limitations. First, due to the retrospective design of this study, some unmeasured confounders such as disease severity might have affected the result, although we had adjusted for all the potential confounders for which we were able to obtain data. Hospital-based studies adjusting for these unmeasured potential confounders are needed to confirm our findings. Second, NS was defined as a new-onset neurological or psychiatric disease after COP. This definition might overestimate the occurrence of NS and dilute the effect of real neurological damage sustained during the poisoning, but it might not affect our conclusions because it should lead to random (non-differential) misclassifications. Third, we did not evaluate the severity of NS because the claim data did not include information on severity. Further study may be necessary to clarify this issue. Fourth, we did not evaluate the association between the duration before HBOT was started after establishing the diagnosis of COP and the occurrence of NS because the claim data did not include information on such durations. Fifth, although this study covered the whole nation, it may not be generalized to other nations due to the difference in race, treatment protocol, and medical resources.

## 5. Conclusions

This nationwide population-based cohort study showed that the risk for NS was higher in the COP patients who received HBOT in comparison with those who did not. Because HBOT reduces mortality, the fact that high-risk survivors tended to develop NS afterwards might contribute to the increase in the risk. Another likely contributing factor was that patients who had risk factors for NS were more likely to receive HBOT. Further prospective studies that include potential confounding factors that we did not measure are warranted to confirm our speculations on the factors contributing to the increased risk for NS.

## Figures and Tables

**Table 1:** Demographic characteristics, underlying comorbidities, monthly income, and concomitant conditions in the patients with COP between 1999 and 2012.

Variable	Total Patients *n* = 24,046 (100.0)	Without HBOT *n* = 17,253 (71.8)	With HBOT *n* = 6793 (28.2)	*p*-Value *
Age (years)	35.7 ± 16.6	36.1 ± 17.2	34.8 ± 14.8	<0.001
Age subgroup (years)				
<20	3382 (14.1)	2509 (14.5)	873 (12.9)	<0.001
20−34	8987 (37.4)	6221 (36.1)	2766 (40.7)	
35−50	7293 (30.3)	5148 (29.8)	2145 (31.6)	
51−64	2996 (12.5)	2223 (12.9)	773 (11.4)	
≥65	1388 (5.8)	1152 (6.7)	236 (3.5)	
Sex				
Female	12,062 (50.2)	8653 (50.2)	3409 (50.2)	0.966
Male	11,984 (49.8)	8600 (49.9)	3384 (49.8)	
Underlying comorbidity				
Hypertension	2815 (11.7)	2216 (12.8)	599 (8.8)	<0.001
Diabetes	1499 (6.2)	1137 (6.6)	362 (5.3)	<0.001
Chronic obstructive pulmonary disease	385 (1.6)	320 (1.9)	65 (1.0)	<0.001
Hyperlipidemia	1967 (8.2)	1486 (8.6)	481 (7.1)	<0.001
Malignancy	643 (2.7)	499 (2.9)	144 (2.1)	<0.001
Coronary artery disease	1358 (5.7)	1067 (6.2)	291 (4.3)	<0.001
Congestive heart failure	392 (1.6)	326 (1.9)	66 (1.0)	<0.001
Liver disease	3307 (13.8)	2428 (14.1)	879 (12.9)	0.022
Renal disease	2445 (10.2)	1804 (10.5)	641 (9.4)	0.013
Connective tissue disease	208 (0.9)	146 (0.9)	62 (0.9)	0.616
HIV infection	61 (0.3)	46 (0.3)	15 (0.2)	0.525
Alcoholism	861 (3.6)	668 (3.9)	193 (2.8)	<0.001
Monthly income (NTD)				
<19,999	17,622 (73.3)	12,670 (73.4)	4952 (72.9)	0.156
20,000−39,999	5122 (21.3)	3629 (21.0)	1493 (22.0)	
≥40,000	1302 (5.4)	954 (5.5)	348 (5.1)	
Concomitant condition				
Suicide	4763 (19.8)	2642 (15.3)	2121 (31.2)	<0.001
Drug poisoning	245 (1.0)	187 (1.1)	58 (0.9)	0.110
Acute respiratory failure	1737 (7.2)	1077 (6.2)	660 (9.7)	<0.001
Acute myocardial injury	54 (0.2)	39 (0.2)	15 (0.2)	0.939
Acute hepatitis	52 (0.2)	39 (0.2)	13 (0.2)	0.602
Acute renal failure	299 (1.2)	167 (1.0)	132 (1.9)	<0.001

COP, carbon monoxide poisoning; HBOT, hyperbaric oxygen therapy; NTD, new Taiwan dollars. Data are presented in n (%). * Comparison between COP patients without and with HBOT.

**Table 2:** Comparison of the risk for NS between patients with COP who received HBOT and who did not by Cox proportional hazard regression and Poisson regression analyses.

Variable	With HBOT			Without HBOT (reference)			Crude HR (95% CI)	AHR (95% CI) *	IRR (95% CI)
	Case	PY	Rate	Case	PY	Rate			
Overall analysis	3043	20,765.3	146.5	5992	64,832.7	92.4	1.5 (1.4–1.6)	1.4 (1.4–1.5)	–
Stratified analysis									
NS subgroup									
Degenerative diseases of the central nervous system	657	28,291.9	23.2	1197	80,595.3	14.9	1.5 (1.3–1.6)	1.5 (1.3–1.6)	–
Psychiatric diseases	2076	23,642.8	87.8	4145	69,828.3	59.3	1.4 (1.3–1.5)	1.3 (1.2–1.4)	–
Other diseases of the nervous system	1461	25,357.5	57.6	2619	75,099.8	34.9	1.6 (1.5–1.7)	1.5 (1.4–1.6)	–
Age (years)									
<20	217	3851.3	56.3	465	12,736.9	36.5	1.5 (13–1.8)	1.4 (1.2–1.7)	–
20−34	1243	8449.4	147.1	2170	24,724.1	87.8	1.6 (1.5–1.7)	1.4 (1.3–1.5)	–
35−50	1060	6093.6	174.0	1999	18,418.8	108.5	1.5 (1.4–1.6)	1.4 (1.3–1.5)	–
51−64	393	1890.4	207.9	871	6226.9	139.9	1.5 (1.3–1.6)	1.4 (1.3–1.6)	–
≥65	130	480.6	270.5	487	2726.2	178.6	1.5 (1.2–1.8)	1.4 (1.2–1.7)	–
Sex									
Female	1566	10,574.5	148.1	3235	33,324.4	97.1	1.5 (1.4–1.6)	1.6 (1.3–1.4)	–
Male	1477	10,190.8	144.9	2757	31,508.3	87.5	1.6 (1.5–1.7)	1.5 (1.4–1.6)	–
Underlying comorbidity									
Hypertension	309	1374.7	224.8	925	5416.5	170.8	1.3 (1.1–1.5)	1.3 (1.1–1.5)	–
Diabetes	167	852.4	195.9	437	2676.5	163.3	1.2 (1.0–1.4)	1.2 (1.0–1.4)	–
Chronic obstructive pulmonary disease	42	104.8	400.7	129	730.3	176.7	2.1 (1.4–2.9)	2.1 (1.4–3.0)	–
Hyperlipidemia	257	1008.0	255.0	577	3700.7	155.9	1.6 (1.4–1.8)	1.5 (1.3–1.8)	–
Malignancy	72	258.7	278.3	184	989.0	186.1	1.4 (1.1–1.9)	1.4 (1.1–1.9)	–
Coronary artery disease	148	621.3	238.2	452	2490.7	181.5	1.3 (1.1–1.6)	1.3 (1.0–1.5)	–
Congestive heart failure	33	130.6	252.7	122	616.9	197.8	1.3 (0.9–1.9)	1.5 (1.0–2.3)	–
Liver disease	446	2059.1	216.6	1037	6424.1	161.4	1.3 (1.2–1.5)	1.3 (1.1–1.4)	–
Renal disease	319	1490.7	214	707	4975.9	142.1	1.5 (1.3–1.7)	1.4 (1.2–1.6)	–
Connective tissue disease	38	121.9	311.7	55	345.1	159.4	1.9 (1.3–2.9)	2.0 (1.2–3.2)	–
HIV infection	9	29.2	308.3	13	118.5	109.7	2.5 (1.1–5.9)	10.3 (2.3–45.8)	–
Alcoholism	115	381.3	301.6	308	1574.3	195.7	1.5 (1.2–1.9)	1.6 (1.2–1.9)	–
Monthly income (NTD)									
<19,999	2322	15,060.2	154.2	4573	47,295.3	96.7	1.5 (1.4–1.6)	1.4 (1.4–1.5)	–
20,000−39,999	586	4516.8	129.7	1142	13,501.0	84.6	1.5 (1.3–1.6)	1.4 (1.2–1.5)	–
≥40,000	135	1188.4	113.6	277	4036.4	68.6	1.6 (1.3–1.9)	1.5 (1.2–1.9)	–
Suicide									
Yes	1181	5664.8	208.5	1305	8639.4	151.1	1.3 (1.2–1.4)	1.3 (1.2–1.4)	–
No	1862	15,100.5	123.3	4687	56,193.3	83.4	1.4 (1.3–1.5)	1.5 (1.4–1.5)	–
Drug poisoning									
Yes	33	116.0	284.4	81	620.2	130.6	1.9 (1.2–2.8)	1.6 (1.0–2.6)	–
No	3010	20,649.3	145.8	5911	64,212.5	92.1	1.5 (1.4–1.6)	1.4 (1.4–1.5)	–
Acute respiratory failure									
Yes	429	1268.5	338.2	440	2281.6	192.9	1.6 (1.4–1.8)	1.5 (1.3–1.8)	–
No	2614	19,496.8	134.1	5552	62,551.1	88.8	1.4 (1.4–1.5)	1.4 (1.3–1.5)	–
Follow-up period									
<2 weeks	597	245.2	2434.7	579	620.5	933.1	2.6 (2.3–2.9)	2.4 (2.1–2.7)	2.6 (2.3–2.9)
2–4 weeks	283	290.6	973.8	308	733.9	419.7	1.7 (1.5–2.0)	1.6 (1.4–1.9)	2.3 (2.0–2.7)
1−6 months	682	2279.1	299.2	1010	6162.6	163.9	1.8 (1.6–2.0)	1.6 (1.4–1.7)	1.8 (1.7–2.0)
6−12 months	283	2871.4	98.6	537	7661.6	70.1	1.7 (0.9–1.2)	1.0 (0.8–1.1)	1.4 (1.2–1.6)
1−2 years	306	5422.4	56.4	816	15,028.2	54.3	1.0 (0.9–1.2)	0.9 (0.8–1.1)	1.0 (0.9–1.2)
2−4 years	416	10,066.8	41.3	1121	28,572.7	39.2	1.0 (0.9–1.1)	0.9 (0.8–1.1)	1.1 (0.9–1.2)
≥4 years	476	20,765.3	22.9	1621	64,832.7	25.0	1.1 (0.9–1.2)	1.0 (0.9–1.2)	0.9 (0.8–1.0)

NS, neurological sequelae; COP, carbon monoxide poisoning; HBOT, hyperbaric oxygen therapy; HR, hazard ratio; AHR, adjusted hazard ratio; CI, confidence interval; IRR, incidence rate ratio; PY, person-years; Rate, NS rate per 1000 person-years; NTD, new Taiwan dollars. * Adjusted for age, sex, underlying comorbidity of hypertension, diabetes, hyperlipidemia, malignancy, coronary artery disease, congestive heart failure, chronic obstructive pulmonary disease, liver disease, renal disease, connective tissue disease, HIV infection, alcoholism, monthly income, suicide, drug poisoning, and acute respiratory failure.

**Table 3:** Independent predictors for NS in patients with COP by Cox proportional hazard regression analysis.

	Patients with COP Who Received HBOT		Patients with COP Who Did Not Receive HBOT	
Variable	Crude HR (95% CI)	AHR (95% CI) *	Crude HR (95% CI)	AHR (95% CI) *
Age (years)				
<20	1	1	1	1
20−34	2.4 (2.1–2.6)	2.3 (2.1–2.5)	2.3 (2.0–2.7)	2.2 (1.9–2.5)
35−50	2.9 (2.6–3.2)	2.6 (2.3–2.9)	2.7 (2.3–3.1)	2.4 (2.0–2.8)
51−64	3.5 (3.1–3.9)	2.8 (2.5–3.2)	3.0 (2.5–3.5)	2.6 (2.2–3.1)
≥65	4.3 (3.8–4.9)	3.4 (2.9–3.9)	3.5 (2.8–4.3)	2.9 (2.2–3.7)
Sex				
Female	1	1	1	1
Male	0.9 (0.9–0.9)	1.2 (1.2–1.3)	1.0 (0.9–1.1)	1.1 (1.0–1.2)
Monthly income (NTD)				
<19,999	1.4 (1.2–1.6)	1.4 (1.3–1.6)	1.3 (1.1–1.6)	1.3 (1.1–1.6)
20,000−39,999	1.2 (1.1–1.4)	1.2 (1.0–1.3)	1.1 (0.9–1.3)	1.1 (0.9–1.3)
≥40,000	1	1	1	1
Underlying comorbidity				
Hypertension	1.8 (1.7–1.9)	1.2 (1.1–1.3)	1.4 (1.2–1.6)	1.0 (0.9–1.2)
Diabetes	1.6 (1.5–1.8)	1.0 (0.9–1.1)	1.2 (1.0–1.4)	0.7 (0.6–0.9)
Chronic obstructive pulmonary disease	1.7 (1.4–2.0)	1.0 (0.8–1.2)	2.0 (1.5–2.7)	1.5 (1.1–2.1)
Hyperlipidemia	1.6 (1.5–1.7)	1.0 (0.9–1.1)	1.5 (1.3–1.7)	1.3 (1.1–1.5)
Malignancy	1.8 (1.5–2.0)	1.2 (1.0–1.4)	1.5 (1.2–1.9)	1.1 (0.9–1.4)
Coronary artery disease	1.8 (1.7–2.0)	1.1 (1.0–1.2)	1.4 (1.2–1.6)	1.0 (0.8–1.2)
Congestive heart failure	1.8 (1.5–2.1)	1.0 (0.8–1.2)	1.4 (1.0–2.0)	0.9 (0.6–1.3)
Liver disease	1.8 (1.6–1.9)	1.3 (1.2–1.4)	1.4 (1.2–1.5)	1.1 (0.9–1.2)
Renal disease	1.5 (1.4–1.6)	1.1 (1.0–1.2)	1.3 (1.2–1.5)	1.1 (1.0–1.2)
Connective tissue disease	1.6 (1.2–2.1)	1.1 (0.9–1.5)	1.7 (1.3–2.4)	1.3 (1.0–1.8)
HIV infection	1.1 (0.6–1.9)	1.0 (0.6–1.7)	1.7 (0.9–3.2)	1.2 (0.6–2.3)
Alcoholism	1.9 (1.7–2.2)	1.4 (1.2–1.5)	1.7 (1.4–2.0)	1.3 (1.1–1.6)
Concomitant condition				
Suicide	1.8 (1.7–1.9)	1.5 (1.4–1.6)	1.6 (1.5–1.7)	1.4 (1.3–1.5)
Drug poisoning	1.4 (1.1–1.7)	1.2 (1.0–1.5)	1.6 (1.2–2.3)	1.5 (1.1–2.1)
Acute respiratory failure	2.0 (1.8–2.2)	1.6 (1.4–1.7)	2.1 (1.9–2.3)	1.7 (1.6–1.9)

NS, neurological sequelae; COP, carbon monoxide poisoning; HBOT, hyperbaric oxygen therapy; HR, hazard ratio; AHR, adjusted hazard ratio; CI, confidence interval; NTD, new Taiwan dollars. * Adjusted for age, sex, underlying comorbidity of hypertension, diabetes, hyperlipidemia, malignancy, coronary artery disease, congestive heart failure, chronic obstructive pulmonary disease, liver disease, renal disease, connective tissue disease, HIV infection, alcoholism, monthly income, suicide, drug poisoning, and acute respiratory failure.

## Supplementary Materials

The following are available online at http://www.mdpi.com/2077-0383/7/10/349/s1, Table S1: Demographic characteristics, underlying comorbidities, monthly income, and concomitant conditions in the patients with COP between 1999 and 2012, Table S2: Comparison of the risk for NS between patients with COP who received HBOT and who did not by Cox proportional hazard regression analysis.

## Abbreviations

HBOT	hyperbaric oxygen therapy
NS	neurological sequelae
COP	carbon monoxide poisoning
NBO	normobaricoxygen
COPD	chronic pulmonary obstructive disease
HIV	Human Immunodeficiency Virus
AHR	adjusted hazard ratio
CI	confidence interval
CO	carbon monoxide
NPD	Nationwide Poisoning Database
NHIRD	National Health Insurance Research Database
ICD-9-CM	International Classification of Diseases, Ninth Revision, Clinical Modification
COHb	carboxyhemoglobin
IRR	incidence rate ratio
ATA	atmosphere absolute

## References

[1] United States Environmental Protection Agency An Introduction to Indoor Air Quality (IAQ). Carbon Monoxide (CO). http://www.epa.gov/iaq/co.html.

[2] CDC (Centers for Disease Control and Prevention) (2005). Unintentional, non-fire-related, carbon monoxide exposures―United States, 2001–2003. MMWR Morb. Morta.l Wkly. Rep..

[3] Prockop L.D., Chichkova R.I. (2007). Carbon monoxide intoxication: An updated review. J. Neurol. Sci..

[4] Ernst A., Zibrak J.D. (1998). Carbon monoxide poisoning. N. Engl. J. Med..

[5] Hampson N.B., Weaver L.K. (2007). Carbon monoxide poisoning: A new incidence for an old disease. Undersea Hyperb. Med..

[6] Hampson N.B., Piantadosi C.A., Thom S.R., Weaver L.K. (2012). Practice recommendations in the diagnosis, management, and prevention of carbon monoxide poisoning. Am. J. Respir. Crit. Care.

[7] Pan Y.J., Liao S.C., Lee M.B. (2010). Suicide by charcoal burning in Taiwan, 1995–2006. J. Affect. Disord..

[8] Wolf S.J., Lavonas E.J., Sloan E.P., Jagoda A.S. (2008). Clinical policy: Critical issues in the management of adult patients presenting to the emergency department with acute carbon monoxide poisoning. Ann. Emerg. Med..

[9] Buckley N.A., Juurlink D.N., Isbister G., Bennett M.H., Lavonas E.J. (2011). Hyperbaric oxygen for carbon monoxide poisoning. Cochrane Database Syst. Rev..

[10] Buckley N.A., Juurlink D.N. (2013). Carbon monoxide treatment guidelines must acknowledge the limitations of the existing evidence. Am. J. Respir. Crit. Care.

[11] Hampson N.B., Piantadosi C.A., Thom S.R., Weaver L.K. (2013). Reply: Carbon monoxide treatment guidelines must acknowledge the limitations of the existing evidence. Am. J. Respir. Crit. Care.

[12] Weaver L.K., Hopkins R.O., Chan K.J., Churchill S., Elliott C.G., Clemmer T.P., Orme J.F., Thomas F.O., Morris A.H. (2002). Hyperbaric oxygen for acute carbon monoxide poisoning. N. Engl. J. Med..

[13] Huang C.C., Ho C.H., Chen Y.C., Lin H.J., Hsu C.C., Wang J.J., Su S.B., Guo H.R. (2017). Hyperbaric oxygen therapy is associated with lower short- and long-term mortality in patients with carbon monoxide poisoning. Chest.

[14] National Health Insurance Research Database. http://nhird.nhri.org.tw/en/index.html.

[15] Weaver L.K. (2009). Carbon monoxide poisoning. N. Engl. J. Med..

[16] Buckley N.A., Isbister G.K., Stokes B., Juurlink D.N. (2005). Hyperbaric oxygen for carbon monoxide poisoning: A systematic review and critical analysis of the evidence. Toxicol. Rev..

[17] Lin J.-N., Lin C.-L., Lin M.-C., Lai C.-H., Lin H.-H., Yang C.-H., Sung F.-C., Kao C.-H. (2015). Risk of leukaemia in children infected with enterovirus: A nationwide, retrospective, population-based, Taiwanese-registry, cohort study. Lancet Oncol..

[18] Lee C.-C., Lee M.-T., Chen Y.-S., Lee S.-H., Chen Y.-S., Chen S.-C., Chang S.-C. (2015). Risk of Aortic Dissection and Aortic Aneurysm in Patients Taking Oral Fluoroquinolone. JAMA Intern. Med..

[19] National Health Insurance Administration, Ministry of Health and Welfare Items for Medical Payments. http://www.nhi.gov.tw/query/query2.aspx.

[20] National Health Insurance Administration, Ministry of Health and Welfare Classification of Diseases. http://www.nhi.gov.tw/webdata/webdata.aspx?menu=18&menu_id=703&webdata_id=1008.

[21] Annane D., Chadda K., Gajdos P., Jars-Guincestre M.C., Chevret S., Raphael J.C. (2011). Hyperbaric oxygen therapy for acute domestic carbon monoxide poisoning: Two randomized controlled trials. Intens. Care Med..

[22] Scheinkestel C.D., Bailey M., Myles P.S., Jones K., Cooper D.J., Millar I.L., Tuxen D.V. (1999). Hyperbaric or normobaric oxygen for acute carbon monoxide poisoning: A randomised controlled clinical trial. Med. J. Aust..

[23] Huang C.-C., Chung M.-H., Weng S.-F., Chien C.-C., Lin S.-J., Lin H.-J., Guo H.-R., Su S.-B., Hsu C.-C., Juan C.-W. (2014). Long-term prognosis of patients with carbon monoxide poisoning: A nationwide cohort study. PLoS ONE.

[24] Thom S.R., Taber R.L., Mendiguren I.I., Clark J.M., Hardy H.M., Fisher A.B. (1995). Delayed neuropsychologic sequelae after carbon monoxide poisoning: Prevention by treatment with hyperbaric oxygen. Ann. Emerg. Med..

[25] Thom S.R. (2002). Hyperbaric-oxygen therapy for acute carbon monoxide poisoning. N. Engl. J. Med..

[26] Weaver L.K., Valentine K.J., Hopkins R.O. (2007). Carbon monoxide poisoning: Risk factors for cognitive sequelae and the role of hyperbaric oxygen. Am. J. Respir. Crit. Care.

[27] Zou J.-F., Guo Q., Shao H., Li B., Du Y.X., Liu M.-F., Liu F.-L., Dai L.-X., Lin H.-J., Su S.-B. (2015). Lack of pupil reflex and loss of consciousness predict 30-day neurological sequelae in patients with carbon monoxide poisoning. PLoS ONE.

[28] Zou J.-F., Guo Q., Shao H., Li B., Du Y.-X., Liu M.-F., Liu F.-L., Dai L.-X., Chung M.-H., Lin H.-J. (2014). A positive Babinski reflex predicts delayed neuropsychiatric sequelae in Chinese patients with carbon monoxide poisoning. Biomed. Res. Int..

[29] Hu H., Pan X., Wan Y., Zhang Q., Liang W. (2011). Factors affecting the prognosis of patients with delayed encephalopathy after acute carbon monoxide poisoning. Am. J. Emerg. Med..

[30] Shen C.-H., Lin J.-Y., Pan K.-T., Chou Y.-C., Peng C.-K., Huang K.-L. (2015). Predicting poor outcome in patients with intentional carbon monoxide poisoning and acute respiratory failure: A retrospective study. J. Med. Sci..

[31] Liu K.Y., Beautrais A., Caine E., Chan K., Chao A., Conwell Y., Law C., Lee D., Li P., Yip P. (2007). Charcoal burning suicides in Hong Kong and urban Taiwan: An illustration of the impact of a novel suicide method on overall regional rates. J. Epidemiol. Commun. Health.

[32] Runyan C.W., Johnson R.M., Yang J., Waller A.E., Perkis D., Marshall S.W., Coyne-Beasley T., MSPH K.S.M. (2005). Risk and protective factors for fires, burns, and carbon monoxide poisoning in U.S. households. Am. J. Prev. Med..

[33] Johnson-Arbor K., Liebman D.L., Carter E.M. (2012). A survey of residential carbon monoxide detector utilization among Connecticut Emergency Department patients. Clin. Toxicol..

[34] Lemstra M., Rogers M., Moraros J. (2015). Income and heart disease: Neglected risk factor. Can. Fam. Physician.

[35] Behera S.K., Winkleby M.A., Collins R. (2000). Low awareness of cardiovascular disease risk among low-income African-American women. Am. J. Health Promot..

