# Supplementary material for: Impact of Hyperbaric Oxygen Therapy on Subsequent Neurological Sequelae Following Carbon Monoxide Poisoning
Source: J Clin Med. 2018 Oct 13;7(10):349. doi: 10.3390/jcm7100349 (PMC6211110; doi:10.3390/jcm7100349)
Supplement: Supplementary file 1 [file jcm-07-00349-s001.pdf]

Supplement Table S1. Demographic characteristics, underlying comorbidities, monthly income, and concomitant conditions in the patients with COP between 1999 and 2012

Variable	Total patients n = 24046	Propensity score matching		
		Without HBOT n = 10144	With HBOT n = 5072	*p*-value*
Age (years)	35.7 ± 16.6	34.8 ± 16.5	34.9 ± 16.1	0.510
Age subgroup (years)				
< 20	3382 (14.1)	1603 (15.8)	800 (15.8)	0.986
20−34	8987 (37.4)	3783 (37.3)	1874 (37.0)	
34−50	7293 (30.3)	2975 (29.3)	1510 (29.8)	
51−64	2996 (12.5)	1315 (13.0)	653 (12.9)	
≥ 65	1388 (5.8)	468 (4.6)	235 (4.6)	
Sex				
Female	12062 (50.2)	5332 (52.6)	2658 (52.4)	0.854
Male	11984(49.8)	4812 (47.4)	3414 (47.6)	
Underlying comorbidity				
Hypertension	2815(11.71)	1068(10.53)	545(10.75)	0.682
Diabetes	1499 (6.2)	582 (5.7)	294 (5.8)	0.883
Chronic obstructive pulmonary disease	385 (1.6)	124 (1.2)	61 (1.2)	0.917
Hyperlipidemia	1967 (8.2)	794 (7.8)	396 (7.8)	0.966
Malignancy	643 (2.7)	219 (2.2)	133 (2.6)	0.073
Coronary artery disease	1358 (5.7)	491 (4.8)	258 (5.1)	0.508
Congestive heart failure	392 (1.6)	131 (1.3)	66 (1.3)	0.960
Liver disease	3307 (13.8)	1294 (12.8)	677 (13.4)	0.306
Renal disease	2405 (10.0)	948 (9.4)	489 (9.6)	0.557
Connective tissue disease	208 (0.9)	96 (1.0)	46 (0.9)	0.812
HIV infection	61 (0.3)	31 (0.3)	15 (0.3)	0.917
Alcoholism	861 (3.6)	344 (3.4)	165 (3.3)	0.655
Monthly income (NTD)				
<19,999	17622 (73.3)	7335 (72.3)	3659 (72.1)	0.962
20,000−39,999	5122 (21.3)	2237 (22.1)	1122 (22.1)	

≥40,000	1302 (5.4)	572 (5.6)	291 (5.7)	
Concomitant condition				
Suicide	4763 (19.8)	571 (5.6)	289 (5.7)	0.862
Drug poisoning	245 (1.0)	8 (0.1)	4 (0.1)	>0.999
Acute respiratory failure	1737 (7.2)	490 (4.8)	278 (5.5)	0.084

COP, carbon monoxide poisoning; HBOT, hyperbaric oxygen therapy; NTD, new Taiwan dollars. Data was presented n (%). *Comparison between patients with COP without and with HBOT.

Supplement Table S2. Comparison of the risk for NS between patients with COP who received HBOT and who did not by Cox proportional hazard regression analysis

	With HBOT			Without HBOT (reference)			Crude HR (95% CI)	AHR (95% CI)*
	Case	PY	Rate	Case	PY	Rate		
Overall analysis	2086 (41.1)	16011.3	130.3	3399 (33.5)	38853.1	87.5	1.4 (1.3−1.5)	1.4 (1.3−1.5)
Stratified analysis								
Age								
< 20	181 (22.6)	3610.8	50.1	293 (18.3)	8052.6	36.4	1.4 (1.1−1.6)	1.4 (1.1−1.6)
20−34	759 (40.5)	5843.2	129.9	1256 (33.2)	15093.0	83.2	1.5 (1.3−1.6)	1.5 (1.3−1.6)
35−50	693 (45.9)	4493.8	154.2	1128 (37.9)	10814.7	104.3	1.4 (1.3−1.6)	1.4 (1.3−1.5)
51−64	324 (49.6)	1583.0	204.7	513 (39.0)	3778.2	135.8	1.5 (1.3−1.7)	1.5 (1.3−1.7)
≥ 65	129 (54.9)	480.5	268.5	209 (44.7)	1114.7	187.5	1.4 (1.1−1.7)	1.4 (1.1−1.7)
Sex								
Female	1100 (41.4)	8547.0	128.7	1906 (35.8)	20986.3	90.8	1.4 (1.3−1.5)	1.4 (1.3−1.5)
Male	986 (40.9)	7464.4	132.1	1493 (31.0)	17866.8	83.6	1.5 (1.4−1.6)	1.5 (1.4−1.6)
Underlying comorbidity								
Hypertension	281 (51.6)	1238.5	226.9	466 (43.6)	2731.4	170.6	1.3 (1.1−1.5)	1.3 (1.1−1.5)
Diabetes	133 (45.2)	689.0	193.0	227 (39.0)	1364.9	166.3	1.2 (0.9−1.4)	1.2 (0.9−1.5)
Chronic obstructive pulmonary disease	38 (62.3)	96.9	392.2	59 (47.6)	297.7	198.1	1.7 (1.1−2.6)	2.2 (1.3−3.4)
Hyperlipidemia	207 (52.3)	799.4	258.9	309 (38.9)	2049.1	150.8	1.6 (1.4−1.9)	1.6 (1.4−2.0)
Malignancy	128 (49.6)	553.0	231.5	215 (43.8)	1255.2	171.3	1.3 (1.1−1.6)	1.3 (1.1-1.7)

Coronary artery disease	66 (49.6)	240.2	274.7	82 (37.4)	494.4	165.9	1.5 (1.1−2.0)	1.5 (1.1−2.2)
Congestive heart failure	33 (50.0)	130.6	252.7	58 (44.3)	268.6	216.0	1.2 (0.8−1.8)	1.5 (0.9−2.4)
Liver disease	331 (48.9)	1557.0	212.6	586 (45.3)	3435.9	170.6	1.2 (1.0−1.4)	1.2 (1.0−1.4)
Renal disease	256 (52.4)	1143.5	223.9	384 (40.5)	2764.7	138.9	1.5 (1.3−1.8)	1.5 (1.3−1.7)
Connective tissue disease	27 (58.7)	85.9	314.4	37 (38.5)	261.3	141.6	2.1 (1.3−3.5)	2.1 (1.2−3.8)
HIV infection	9 (60.0)	29.2	308.3	10 (32.3)	85.1	117.5	2.4 (0.9−5.9)	24.5 (3.6−166.0)
Alcoholism	100 (60.6)	333.4	300.0	171 (49.7)	819.5	208.7	1.4 (1.1−1.8)	1.5 (1.2−1.9)
Monthly income (NTD)								
<19,999	1580 (43.2)	11508.2	137.3	2555 (34.8)	27785.9	92.0	1.4 (1.3−1.5)	1.4 (1.3−1.5)
20,000−39,999	400 (35.7)	3460.4	115.6	680 (30.4)	8576.3	79.3	1.4 (1.2−1.6)	1.4 (1.2−1.5)
≥40,000	106 (36.4)	1042.8	101.7	164 (28.7)	2491.0	65.8	1.5 (1.2−1.9)	1.5 (1.2−1.9)
Suicide								
Yes	224 (56.0)	910.8	246.0	374 (47.7)	2115.0	176.8	1.3 (1.1−1.6)	1.3 (1.1−1.6)
No	1862 (39.9)	15100.5	123.3	3025 (32.3)	36738.2	82.3	1.4 (1.3−1.5)	1.4 (1.3−1.5)
Drug poisoning								
Yes	31 (57.4)	111.8	277.3	55 (46.6)	371.6	148.0	1.6 (1.1−2.6)	1.6 (0.9−2.8)
No	2055 (41.0)	15899.5	129.3	3344 (33.4)	38481.5	86.9	1.4 (1.3−1.5)	1.4 (1.3−1.5)
Acute respiratory failure								
Yes	220 (64.9)	629.5	349.5	213 (37.3)	1310.0	162.6	1.8 (1.5−2.2)	1.8 (1.5−2.2)
No	1866 (39.4)	15381.8	121.3	3186 (33.3)	37543.2	84.9	1.4 (1.3−1.5)	1.4 (1.3−1.5)
Follow-up period								
<2 weeks	410 (8.1)	183.5	2233.9	320 (3.2)	365.3	875.9	2.5 (2.2−2.9)	2.5 (2.2−2.9)
2 weeks−4 weeks	188 (3.7)	217.5	864.4	168 (1.7)	431.9	389.0	1.7 (1.4−2.1)	1.7 (1.4−2.1)

1−6 months	426 (8.4)	1745.0	244.1	530 (5.2)	3653.9	145.1	1.6 (1.4−1.8)	1.6 (1.4−1.8)
6−12 months	197 (3.9)	2213.9	89.0	300 (3.0)	4546.0	66.0	1.0 (0.8−1.2)	1.0 (0.8−1.2)
1−2 years	230 (4.5)	4209.8	54.6	471 (4.6)	8940.9	52.7	0.9 (0.8−1.1)	0.9 (0.8−1.1)
2−4 years	301 (5.9)	7798.3	38.6	665 (6.6)	17106.6	38.9	0.9 (0.8−1.1)	0.9 (0.8−1.1)
≥4 years	334 (6.6)	16011.3	20.9	945 (9.3)	38853.1	24.3	1.0 (0.9−1.1)	1.0 (0.9−1.11)

NS, neurological sequelae; COP, carbon monoxide poisoning; HBOT, hyperbaric oxygen therapy; AHR, adjusted hazard ratio; CI, confidence interval; NTD, new Taiwan dollars. Data was presented n (%). *Adjusted for age, sex, underlying comorbidity of hypertension, diabetes, chronic obstructive pulmonary disease, hyperlipidemia, malignancy, coronary artery disease, congestive heart failure, liver disease, renal disease, connective tissue disease, HIV infection, alcoholism, monthly income, suicide, drug poisoning, and acute respiratory failure.
